# Factors associated with flexible cystoscope longevity: an analysis of supplier and health service datasets

**DOI:** 10.1111/bju.70133

**Published:** 2025-12-23

**Authors:** Joseph B. John, Robert Feasey, Ranan Dasgupta, Tim W.R. Briggs, John S. McGrath, William K. Gray

**Affiliations:** ^1^ University of Exeter Medical School University of Exeter Exeter UK; ^2^ Department of Urology Royal Devon University Healthcare NHS Foundation Trust Exeter UK; ^3^ Getting It Right First Time Programme NHS England London UK; ^4^ KARL STORZ Endoscopy (UK) Ltd Slough UK; ^5^ Imperial College Healthcare NHS Trust London UK; ^6^ Royal National Orthopaedic Hospital, Stanmore London UK; ^7^ Urology Department North Bristol NHS Foundation Trust Bristol UK

**Keywords:** flexible cystoscopy, bladder cancer, sustainability, circular economy, design for life, reusable medical devices

## Abstract

**Objectives:**

To investigate the associations between how reusable flexible cystoscopes (FCs) are managed during their functioning lifetime and their longevity.

**Patients and Methods:**

This was an exploratory retrospective analysis of administrative data collected by a medical supplies company (KARL STORZ Endoscopy (UK) Ltd) on FC usage linked at a National Health Service (NHS) hospital trust level to data from NHS England's Hospital Episodes Statistics dataset on the number of FCs performed each year in the NHS in England. Number of uses before failure (defined as user‐determined need for FC return to the supplier) were analysed descriptively and using a negative binomial regression model.

**Results:**

Data were available for 1918 FCs across 70 hospitals. The median (interquartile range) number of uses and min of use before failure were 58 (20–147.75) and 706.5 (208.25–1718), respectively. Eighty‐five percent of returned FCs were exchanged (i.e. replaced with a new FC), at a median of 66 uses. The two most common reasons for failure – damage to the working channel and control handle housing – were observed in 76.3% and 63.8% of returned FCs, respectively. A greater number of uses before failure was significantly associated with recency, same‐site same‐complex decontamination, on‐site endoscopic specialist availability, decontamination in a general endoscopy unit, and drying cabinet or bowl storage rather than vacuum packaging. Top‐quintile‐volume units were associated with a significantly higher number of uses before failure, however, there was otherwise no clear independent volume–longevity association.

**Conclusion:**

This exploratory analysis generates mechanistically plausible hypotheses regarding factors that could promote FC longevity. These findings are of relevance as we seek to understand how to optimise the cost, resilience and environmental sustainability of healthcare. A prospectively designed study could investigate whether there is a causal link between the key factors identified and longevity of FC usage.

**Abbreviations**
FC: flexible cystoscope
HES: Hospital Episodes Statistics
IQR: interquartile range
IRR: incident rate ratio
OES: on‐site endoscopic specialist
OPCS‐4: Office of Populations Censuses and Surveys Classification of Interventions and Procedures version 4

## Introduction

Worldwide, there are nearly half a million new bladder cancer diagnoses annually [1]. Flexible cystoscopy is the first‐line investigation for suspected bladder cancer and is the main investigation carried out in post‐treatment surveillance for most patients [2]. Additional indications for flexible cystoscopy include treating smaller bladder tumours, detrusor muscle botulinum toxin injections for bladder overactivity, and removal of ureteric stents [3, 4, 5]. In the UK, over 300 000 flexible cystoscopies are performed annually [6].

Despite numerous studies comparing different reusable and single‐use flexible cystoscopes (FCs) in terms of their clinical performance, cost and environmental sustainability, no large multicentre studies have investigated average FC longevity (the amount of usage in their working lifespan) or how best to promote this [7, 8, 9, 10, 11]. Current understanding about how to promote FC longevity is largely derived from first principles, experience, and single‐centre studies, with derived expertise and hypotheses guiding the operations of companies and urological care providers [12]. Understanding how to promote FC longevity is important because FC breakage requiring repair or replacement has implications for cost, service continuity, patient care and the environment [13, 14]. Current single‐centre data describe FC longevity ranging widely from a mean of 135 (for fibreoptic FCs) to 3920 uses before irreparable breakage [7, 12].

Several publications describe flexible ureteroscope longevity, which is considerably lower than that reported for FCs, with reported lifespans ranging from 9 to 79 uses before breakage [15, 16]. In 2024, Thöne et al. published a comparison of reusable and single‐use flexible ureteroscopes that offered insights into how different factors can influence the performance of urological endoscopes from an environmental perspective [17]. The number of uses achieved before irreparable damage was a key factor, with a ‘break‐even’ point for greenhouse gas emissions of 7.9 uses for their modelled base case in comparison with single‐use ureteroscopes. Maximising ureteroscope longevity is also acknowledged as being important from a cost perspective [15].

Beyond urology, gastrointestinal endoscopes have been shown to frequently last for >1000 uses, although smaller‐calibre gastrointestinal endoscopes have been shown to break sooner [18, 19]. One study demonstrated that the training of decontamination staff in endoscope handling reduced the gastrointestinal endoscope breakage rate [20].

In this multicentre exploratory study, we assessed the associations between how FCs are managed during their functioning lifetime and their longevity. To do this we used administrative healthcare data and commercial records and describe the variation in the longevity of FCs across hospitals and the main causes of failure. We assessed the association between longevity and prospectively hypothesised mechanisms of failure, as described in the literature.

## Methods

### Study Design

This was an exploratory retrospective analysis of administrative data collected by a medical supplies company (KARL STORZ Endoscopy (UK) Ltd) on the use of FCs linked at an NHS hospital trust level to data from NHS England's Hospital Episodes Statistics (HES) dataset on the number of flexible cystoscopies performed each year in the NHS in England.

### Ethics

The presentation of data follows current NHS England guidance for use of administrative data for research purposes. This was an analysis of routinely collected administrative/commercial data. The primary data were provided at a FC level. No healthcare patients were involved in the study and the data do not relate to individual patients. Ethical approval was not required.

### Data Collection

Data on the number of FC uses before failure at a device level, mechanism of failure and the endoscope management characteristics of the end user hospital were provided by KARL STORZ Endoscopy (UK) Ltd, covering 65 months from 1 March 2019 to 31 July 2024. Data were available for the NHS and independent sector (private) hospitals across the UK which the company provides with endoscopes for use in humans. A KARL STORZ Endoscopy (UK) Ltd representative (R.F.) provided the data to the NHS England team (J.B.J., W.K.G.) and added context and insight to the data and analysis though a series of conversations. Details on the data provided are given below.

Data on trust volumes for flexible cystoscopy procedures were extracted from the HES dataset for the same time period. Data were only available for NHS hospitals in England. Cystoscopy was identified using the Office of Populations Censuses and Surveys Classification of Interventions and Procedures version 4 (OPCS‐4) code M45‐(diagnostic endoscopic examination of bladder). OPCS‐4 codes are unable to distinguish between rigid cystoscopes and FCs, therefore, a broader definition was used. The care records for admitted patients and outpatient records were searched, emergency and elective admissions were included, and the codes were identified when present anywhere in the procedural record.

### Outcomes of Interest

The primary outcome of interest was the number of patient uses of the FC before it failed. Failure was defined as a user‐determined need for the FC to be returned to the supplier due to a perceived requirement for repair or exchange. This definition included those FCs that were then assessed and deemed by the supplier to have no defect and were returned to the user organisation for further use. A sensitivity analysis was conducted which excluded the FCs that were returned to the supplier where no defect was detected.

On return to the manufacturer, FCs are sent to an examination and test centre in the UK, where any repairs that can be performed are carried out. Viable FCs are then returned to the user. FCs that cannot be repaired are sent from the UK to Germany for further inspection before disposal.

The secondary outcome was the total use time before failure. The mean use time per patient‐use was also calculated.

### Hospital Level Variables of Interest

Data were available at a hospital or hospital trust level. NHS hospitals are run by trusts and each trust manages between one and four large (e.g. district general) hospitals. A range of variables of perceived relevance were considered when assessing factors associated with longevity:

1. Cleaning location in relation to end user (hospital) location: on‐site and same complex as end user; on‐site and different complex to end user; or off‐site.
2. Type of decontamination unit: dedicated urology department decontamination unit; general endoscopy decontamination unit; or general hospital sterilisation and decontamination unit.
3. Method of storage between procedures: drying cabinet; vacuum packed; or bowl and re‐washed (i.e. cystoscope stored in a bowl after use and repeat decontamination performed immediately before the next use).
4. On‐site endoscopic specialist (OES) available. An OES is a dedicated member of KARL STORZ staff included within a Managed Surgical Facility contract. The OES assumes responsibility for equipment management and ensures correct care and equipment handling, undertakes proactive maintenance and delivers local training. OESs aim to increase equipment utilisation and minimise the need for repairs and downtime [21]: yes or no.
5. Frequency of staff decontamination training: once per year; twice per year; or more than twice per year.
6. Whether the hospital was part of the NHS or independently funded.
7. The devolved health system in which the hospital was located (England, Wales, or Scotland).
8. For hospitals in England only, NHS hospital trust cystoscopy mean annual case volume across the 5‐year study period. Given the exploratory nature of the analysis, volumes were categorised for analysis. Categories were chosen such that the category boundaries were meaningful and such that there were broadly similar numbers of patients in each category. Based on exploration of the data distribution, the following categories were chosen: 1–999; 1000–1999; 2000–2999; 3000–3999; and ≥4000 procedures per annum.

### Other Data Collected

Data were also available on the following:

1. The date the FC was returned to the manufacturer.
2. Whether the FC was returned to the end user without repair, repaired, or exchanged.
3. The result of a working channel leak test: pass or fail.
4. The high‐level reason for failure: wear and tear; mechanical damage; physical damage; damage during cleaning; or fluid ingress.
5. The specific failure: chemical corrosion, damage to examination shaft, damage to supply shaft, damage to strain relief, damage to programming unit, poor image quality, damage to shutter function, damage to buttons, damage to light transmission unit, damage to lenses, damage to adhesive rings, damage to angulation cover, external damage, flooding, damage to supply plug, damage to distal head, damage to working channel, damage to control handle housing, damage to temperature indicator, damage to vertebrae, damage to deflection mechanisms.

**Fig. 1:**
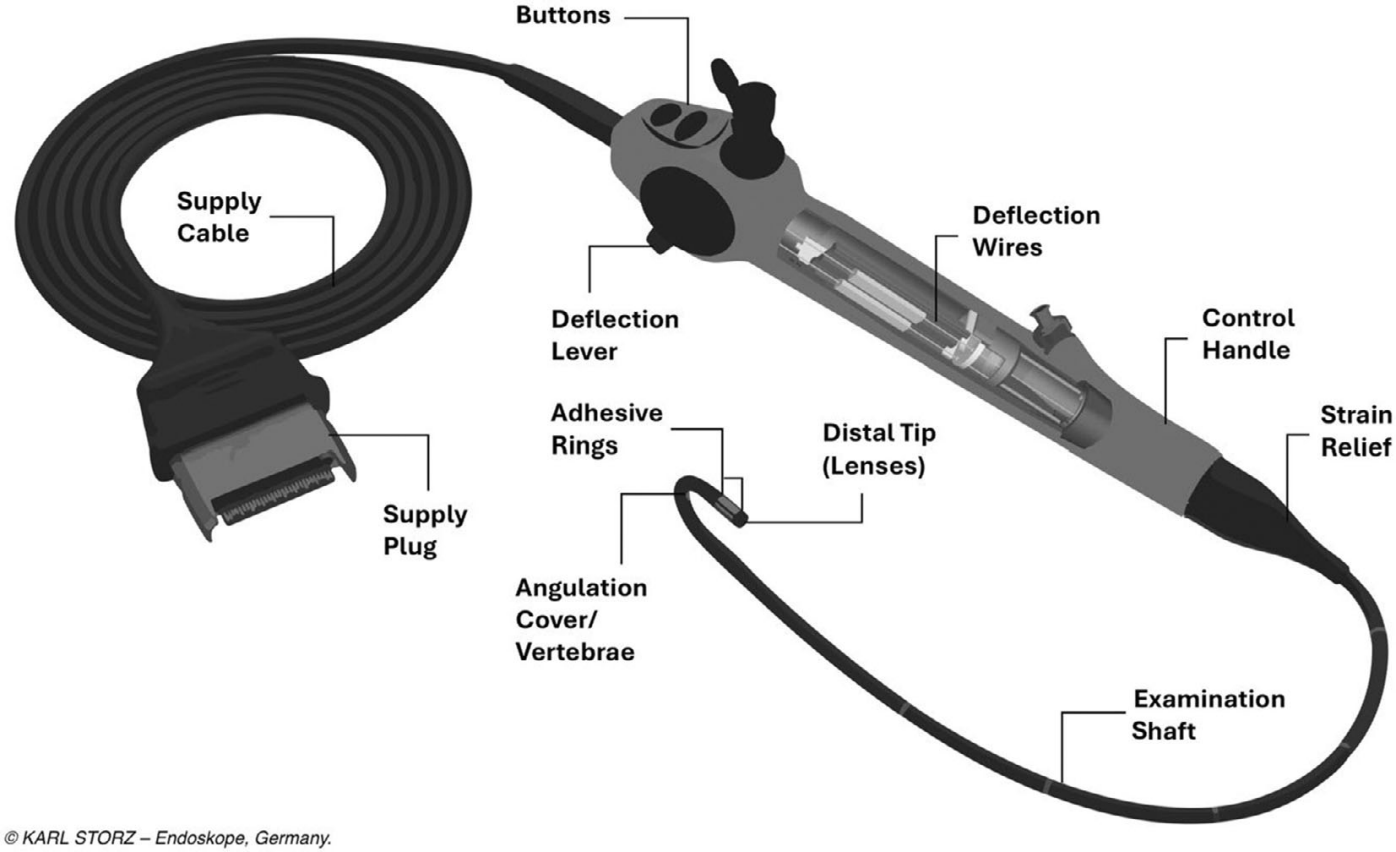
Reusable flexible cystoscope with labelled components.

Figure 1 is an illustration of a reusable FC with components labelled.

### Data Analysis

Data were analysed within a secure encrypted server controlled by NHS England. Analysis within this secure environment took place using standard statistical software: Microsoft Excel (Microsoft Corp, Redmond, WA, USA) and Stata (StataCorp LLC, College Station, TX, USA).

In descriptive analysis, data were categorised as detailed above and summarised in terms of frequency and percentage. The outcomes ‘number of uses prior to failure’ and ‘number of min prior to failure’ were non‐normally distributed, with the majority of the data clustered at the lower end of the data range and a long tail of larger values. As such, these data were described using median and interquartile range (IQR) and modelled using a multivariable negative binomial regression model. The findings are presented in terms of incident rate ratio (IRR) with 95% CIs and significance tests (*P* values) reported. The IRR values should be interpreted as mutually adjusted associations and not causative. Data on hospital volume were only available for hospitals in England, and were therefore added to the models as an additional item and reported separately.

## Results

**Fig. 2:**
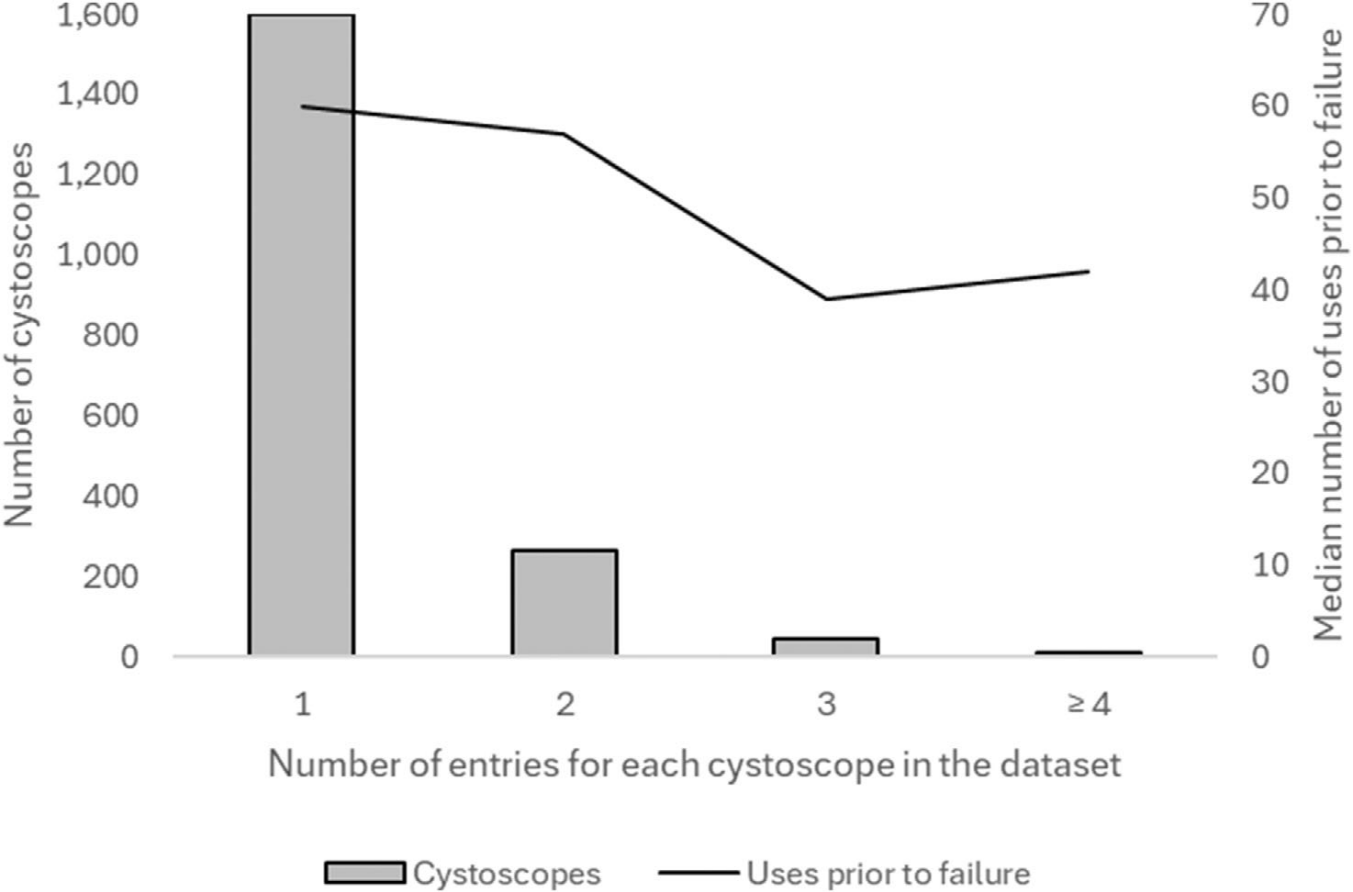
Number of entries in the dataset and median uses prior to failure for individual flexible cystocopes.

Data were available for 1918 flexible cystoscopies across 70 hospitals. The median (IQR) number of FC uses and total minutes of use prior to failure were 58 (20–147.75) and 706.5 (208.25–1718), respectively. Considering only FCs that were exchanged (i.e. not returned to the customer with or without repair), the median number of uses before failure was 66. The median (IQR) number of min per use was 11.8 (7.2–17.4). Summary data on the number of patient uses and the median times to failure categorised according to the variables of interest are presented in Table 1. NHS use dominated and almost 90% of the data were for patients seen in hospitals in England. The median number of uses prior to failure over each year of the study period is also shown in Table 1. Uses and min until failure have both increased over time (2019: 49 uses and 411 min; 2024: 75.5 uses and 925 min). Over 80% of FCs were exchanged for a new one, suggesting that they were irreparable at this point. The number of entries for each individual FC in the dataset and median number of uses until failure for each entry line are shown in Fig. 2. Over 80% of FCs appeared only once in the dataset. Eight FCs appeared four times, two FCs appeared five times, and one FC appeared six times. These were combined for analysis because of low numbers.

**Table 1:** Summary of flexible cystoscope use and characteristics of the hospitals included in the study.

	Number of FCs (%)	Median number of uses prior to failure (IQR, range)	Range of number of uses prior to failure
Type of hospital			
Private	165 (8.6)	24 (10–60)	1–294
NHS	1753 (91.4)	64 (22–156)	1–1118
Hospital location			
England	1700 (88.6)	55 (18–139)	1–1118
Wales	125 (6.5)	128 (47–278)	2–881
Scotland	93 (4.8)	68 (35–181)	2–656
Cleaning location			
Off‐site	70 (3.6)	50 (24–91)	1–247
On‐site and different complex	1149 (59.9)	52 (18–132)	1–773
On‐site and same complex	699 (36.4)	72 (24–165)	1–1118
Decontamination			
Dedicated urology decontamination unit	67 (3.5)	30 (11–113)	1–438
General endoscopy decontamination unit	1556 (81.1)	57.5 (19–145)	1–1118
General hospital sterilisation and decontamination unit	295 (15.4)	68 (27–168)	2–656
Storage			
Vacuum packed	591 (30.8)	36 (13–100)	1–773
Drying cabinet	545 (28.4)	71 (26–165)	1–1118
Bowl and rewashed	782 (40.8)	75 (27–162)	1–881
On‐site endoscopic specialist available			
No	1805 (94.1)	56 (18–140)	1–1118
Yes	113 (5.9)	127 (47–250)	4–635
Frequency of staff decontamination training (32 missing values)			
Once per year	1268 (67.0)	61 (20–149.5)	1–1118
Twice per year	383 (20.2)	66 (20–173)	1–634
More than twice per year	241 (12.7)	42 (14–113)	1–671
Management of endoscope by manufacturer on return			
No defect identified, returned to user without intervention	215 (11.2)	41 (12–92)	1–439
Repaired and return to user	77 (4.0)	14 (6–52)	1–209
Exchanged, user provided with a new endoscope	1626 (84.8)	66 (23–158)	1–1118

FC, flexible cystoscope; IQR, interquartile range.

**Fig. 3:**
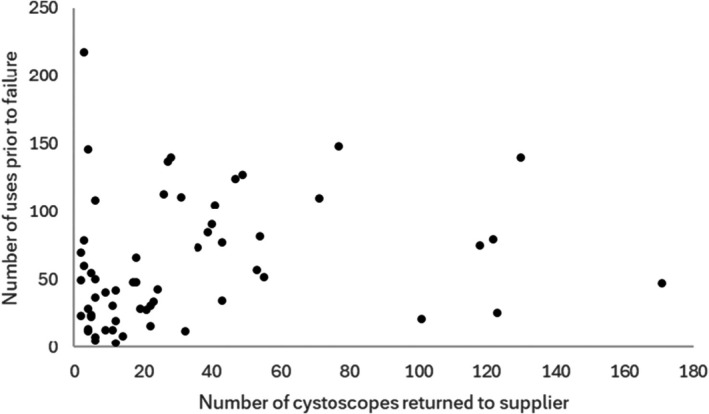
Association between number of flexible cystocopes returned to supplier and median number of uses prior to failure for each hospital.

For the 59 hospitals with data for two or more FCs, the variation in median number of uses until failure relative to the number of devices returned to the supplier for each hospital is shown in Fig. 3; this association was tested using Spearman's correlation coefficient. At a hospital level, there was a significant positive correlation between the number of devices returned to the supplier and the number of uses until failure (Spearman's *r* = 0.323, *P* = 0.013), suggesting a relationship between higher volumes of FC procedures conducted and greater longevity of the FC. For hospitals returning an above‐the‐median (*n* = 19) number of FCs, the median number of uses prior to failure was 77 and for hospitals returning an at‐ or below‐the‐median number of FCs, the median number of uses prior to failure was 34.

The multivariable models of different variables describing each unit and their FC use and management in relation to the number of uses prior to failure are summarised in Table 2. After adjusting for covariates, a higher number of uses prior to failure was significantly associated with the following: being an NHS rather than a private hospital; recency; cleaning carried out on the same site and complex as the procedure; and having an OES available. Decontamination in a general endoscopy unit was associated with the highest number of uses prior to failure, followed by decontamination in a general hospital decontamination and sterilisation unit, with decontamination in a dedicated urology unit being associated with a lower number of uses prior to failure. Storage of the FC in a drying cabinet or bowl was associated with a higher number of uses than storage in a vacuum pack. A greater number of uses prior to failure was also associated with less frequent staff training. After adjusting for covariates, there was no difference in number of uses prior to failure across England, Wales or Scotland. This analysis was repeated after exclusion of the FCs returned to the supplier where no defect was detected. The model is summarised in Table S1 and is very similar to that identified for the primary analysis, although the type of decontamination unit was no longer a significant predictor.

**Table 2:** Multivariable modelling of factors associated with number of uses prior to failure.

Characteristic	IRR (95% CI), significance
Type of hospital	
Private	1 (reference)
NHS	2.42 (2.01–2.92), *P* < 0.001
Hospital location	
England	1 (reference)
Wales	1.01 (0.76–1.34), *P* = 0.950
Scotland	1.05 (0.80–1.39), *P* = 0.707
Year of return to manufacturer	
2019	1 (reference)
2020	1.13 (0.95–1.35), *P* = 0.166
2021	1.32 (1.11–1.56), *P* = 0.001
2022	1.40 (1.18–1.65), *P* < 0.001
2023	1.43 (1.21–1.69), *P* < 0.001
2024	1.42 (1.17–1.73), *P* < 0.001
Cleaning location	
Off‐site	1 (reference)
On‐site and different complex	1.27 (0.96–1.67), *P* = 0.090
On‐site and same complex	1.83 (1.36–2.47), *P* < 0.001
Decontamination	
Dedicated urology decontamination unit	1 (reference)
General endoscopy decontamination unit	1.94 (1.45–2.6), *P* < 0.001
General hospital sterilisation and decontamination unit	1.59 (1.15–2.21), *P* = 0.005
Storage	
Vacuum packed	1 (reference)
Drying cabinet	1.21 (1.01–1.44), *P* = 0.034
Bowl and rewashed	1.22 (1.07–1.39), *P* = 0.003
On‐site endoscopic specialist available	
No	1 (reference)
Yes	1.77 (1.31–2.39), *P* < 0.001
Frequency of staff decontamination training	
Once per year	1 (reference)
Twice per year	0.87 (0.75–1.01), *P* = 0.061
More than twice per year	0.72 (0.6–0.85), *P* < 0.001
England only additional variable	
Annual hospital trust volume	
1st (lowest) volume category	1 (reference)
2nd volume category	0.88 (0.62–1.25), *P* = 0.473
3rd volume category	0.87 (0.69–1.1), *P* = 0.256
4th volume category	0.76 (0.59–0.99), *P* = 0.044
5th (highest) volume category	1.93 (1.54–2.42), *P* < 0.001

1st (lowest) volume category < 1000 annual cases; 2nd volume category = 1000–1999 annual cases; 3rd volume category = 2000–2999 annual cases; 4th volume category = 3000–3999 annual cases; 5th (highest) volume category ≥ 4000 annual cases.

IRR, incident rate ratio.

Data on hospital trust annual procedure volume for hospitals in England were added to the model as an additional variable and the analysis was rerun. The model outcomes for the volume categories after adjusting for all other variables are shown in Table 2. Relative to trusts with an annual volume of <1000, trusts with ≥4000 cystoscopies per annum had a significantly higher number of uses prior to failure, with a near doubling of the IRR. In contrast, the second highest volume category (3000–3999 procedure per annum) had significantly fewer uses prior to failure compared to the <1000 procedures category, although the association in this case was relatively weak.

The models for the outcome ‘min of use prior to failure’ are shown in Table S2 and show a similar pattern of associations to that seen for number of uses prior to failure.

Data on the high‐level reason for failure, the specific reason for failure, and the result of leak testing are summarised in Table 3. Over three‐quarters of FCs returned to the supplier had damage to the working channel and almost two‐thirds had damage to the control handle housing. Angulation cover, distal head, supply plug and adhesive ring damage, and poor image quality were other faults noted for over 20% of FCs. The multivariable models were rerun, looking at the number of uses until failure for each of the top seven specific reasons for failure and the findings are presented in Table S3. Across all seven specific reasons for failure, FCs used in NHS hospitals had greater longevity than in private hospitals. The year‐on‐year improvement in longevity was not apparent for FCs with working channel and control handle housing damage. On‐site decontamination was more obviously associated with longevity for FCs with working channel, angulation cover and supply plug damage, and use of a dedicated endoscopy decontamination unit. Having an OES at the hospital site was associated with longevity for FCs with working channel, angulation cover and distal head damage. Storage in a bowl or drying cabinet was associated with longevity for FCs with working channel and control handle housing damage. Across all reasons for failure, there was no evidence that training more frequently than once per year was beneficial.

**Table 3:** Symptoms and mechanisms of failure.

	FCs (*n* = 1918)
Leak test failed, *n* (%)	1311 (68.4), 2 missing values
High‐level reasons for failure, *n* (%)	
Physical damage	900 (46.9)
Mechanical damage	458 (23.9)
Damage during cleaning	44 (2.3)
Fluid ingress	123 (6.4)
Damage during cleaning	44 (2.3)
Wear and tear	0 (0)
Specific reason for failure, *n* (%)	
Damage to working channel	1461 (76.3), 2 missing values
Damage to control handle housing	1221 (63.8), 3 missing values
Damage to angulation cover, external	483 (45.2), 850 missing values
Damage to supply plug	624 (32.7), 8 missing values
Poor image quality	472 (24.7), 5 missing values
Damage to adhesive rings	463 (24.2), 5 missing values
Damage to distal head	418 (21.9), 8 missing values
Damage to lenses	313 (16.3), 2 missing values
Damage to deflection mechanisms	257 (13.5), 9 missing values
Damage to examination shaft	240 (12.5), 4 missing vales
Damage to shutter function	178 (9.3), 11 missing values
Flooding	68 (6.4), 850 missing values
Damage to vertebrae	95 (5.0), 4 missing values
Damage to temperature indicator	87 (4.6), 8 missing values
Damage to light transmission unit	65 (3.4%), 8 missing values
Damage to supply shaft	63 (3.3), 3 missing values
Damage to buttons	24 (1.3), 7 missing values
Damage to programming unit	23 (1.2%), 4 missing values
Chemical corrosion	14 (0.7), 9 missing values
Damage to strain relief	8 (0.4), 4 missing values

FC, flexible cystoscope.

## Discussion

Our exploratory study is one of very few reports on the longevity of reusable FCs. We identified factors associated with FC longevity, including on‐site same‐complex decontamination, decontamination in a general endoscopy unit, having an OES on site, avoiding vacuum pack storage, and NHS practice. Over 80% of FCs were exchanged for a new one after failure, with fewer than 5% repaired and returned to the user. This low rate of repair, also outlined in Fig. 2, demonstrates the importance of careful equipment handling; any breakage is likely to be irreparable and to require cystoscope replacement, incurring high environmental and financial costs. Our observations could also inform future FC design improvements, which might include incorporating greater device modularity to enable replacement of components, and research to improve the durability of commonly damaged materials [22]. Design and management improvements can help to achieve the goals of the UK Design for Life programme and similar initiatives [14, 22, 23]. This is particularly relevant across high‐income health services, in which the prevailing trend is towards growing use of disposable medical equipment, and also increasing costs and surveillance intensity for non‐muscle‐invasive bladder cancer, specifically [24, 25].

In our study, the median number of FC uses before failure was 58. This is lower than the median of 135 uses of fibreoptic FCs reported by McGill et al. and the median of 207 uses between repairs (equivalent to our working definition of failure) and 3920 uses before exchange reported by Kemble et al. [7, 12]. However, our reported upper interquartile value was 147.75, suggesting that around one quarter of hospitals in our study had average use rates comparable to those reported in single‐centre studies. The relatively wide variation in median number of uses before failure across hospitals in our study highlights a probable opportunity to improve FC longevity in many units. If all hospitals were to reach the longevity represented by our reported upper quartile, the number of individual FCs deployed across the UK each year would fall substantially. The inter‐hospital variation in how FCs are managed and the noted associations between various practices and longevity identifies potential ways in which some of the observed variation in longevity may be modifiable.

Most of the associations with longevity identified during analysis have a plausible mechanism. On‐site same‐complex decontamination will eliminate the potential for damage incurred during transport [26]. Supplier support through OES availability might further enhance staff knowledge about FC handling and decontamination and proactively identify issues that could cause FC damage. Decontamination in a general endoscopy unit means that staff with higher‐volume decontamination experience will generally reprocess FCs, although it should be noted that this association was diminished on sensitivity analysis (Table S1). Higher‐volume clinical practices might lead to more careful FC handling, resulting in less frequent in‐use damage. Avoidance of vacuum packing can circumvent having to coil FCs in a way that might stress the component materials, such as the working channel and angulation cover – two of the most common reasons for failure. Of note, however, a significant difference between vacuum packing and drying cabinet use was not identified in relation to damage to working channel or angulation cover, although a difference was observed in comparison to the bowl‐and‐rewash storage method.

The negative association between the number of uses prior to failure and staff training frequency could have several explanations. More frequent training may reflect relatively high turnover of staff in some decontamination units, with training given to new starters who have low levels of experience in reprocessing FCs. It may also reflect units reacting to high FC breakage rates by arranging additional staff training. Data on why training was arranged, and for which staff groups, would provide additional insight.

We emphasise that our findings should be interpreted as associations only, and not as causative. The data on the relationship between volume of cystoscopies performed and longevity, although triangulated using two methods (Figs 2 and 3), should be interpreted with caution and should perhaps be a signal for further dedicated study prior to drawing firm conclusions. Nevertheless, the observed effect sizes in most cases are large, especially given that the decontamination data were recorded at a hospital rather than an individual FC level. NHS hospitals with on‐site decontamination in a dedicated endoscopy unit, with OES availability, and with storage in a bowl or drying cabinet appear likely to realise the highest median number of uses prior to failure, and this approach may represent a ‘gold standard’. Prospective collection of more detailed FC‐level decontamination data, even if for a small set of FCs, will shed additional light on where our findings have external validity and whether practice recommendations can be made with greater certainty. A further study to assess causality could be conducted across units currently demonstrating particularly low longevity and moderate or high FC return rates (Fig. 3). Improvements here could result in the greatest environmental and financial benefits because absolute incremental improvements in longevity would reduce the embodied carbon footprint and cost of supplying each FC per unit of clinical activity substantially [27].

Our data cover FC use across an entire country, with 70 hospital sites included in the dataset. However, the data were from a single supplier and endoscopes from another supplier might have a different profile with regards to longevity and amenability to repair. Nevertheless, many of the associations noted are likely to be similar across all FC manufacturers, given the similarity in design, use and decontamination practices.

The primary data source was at an FC level rather than a patient level. Likewise, data on decontamination were available at a hospital rather than a patient level and represent the practice of the hospital as a whole, rather than reflecting how an individual FC was managed. Patient‐level data, from use through to decontamination, could have allowed us to comment on and adjust our models for case mix, surgeon characteristics and specific decontamination details for each use. Breakdown of usage of instruments such as forceps (for stent removal) and laser fibres (for tumour ablation) would also be informative since these might accelerate channel damage. The increasing use of FC‐delivered interventions, particularly treatment for non‐muscle‐invasive bladder cancer, means that understanding how such applications influence longevity is important [5, 6, 28, 29]. Although collection of such data would be complex, time‐consuming and costly on a large scale, it might provide additional actionable insights in relation to longevity. We are conscious that the OES role and function may be unique to KARL STROZ Endoscopy (UK) Ltd, and not applicable to other similar datasets. However, excluding it as a variable would have risked biasing our findings. Lastly, as with any study using administrative data, the information noted above were lacking and, as such, it is possible that unmeasured confounding could bias our findings.

In conclusion, our study describes FC longevity, its variation across a nation, and how different approaches to FC use and management are associated with longevity. This study substantially adds to the limited pre‐existing evidence base and most of the identified associations carry mechanistic plausibility, although causation cannot be assigned using these data. These findings are of relevance as we seek to understand how to optimise the cost, resilience and environmental sustainability of healthcare, with considered use of long‐lasting medical devices seen as a priority. A prospectively designed study could further investigate whether key factors identified in this analysis do promote FC longevity.

## Author Contributions

J.B.J., W.K.G. and J.S.M. designed this study; R.F. provided the data and informed data cleaning by J.B.J. and W.K.G.; J.B.J. and W.K.G. analysed the data and wrote the first draft of the manuscript; J.S.M., R.F., R.D. and T.W.R.B. critically reviewed the manuscript. All authors approved the manuscript for submission.

## Disclosure of Interests

J.B.J., W.K.G. and J.S.M. are funded by the Sustainable Health Systems Hub: Creating Sustainable Health and Social Care Pathways, based at the University of Exeter, which is supported by UK Research and Innovation Building a Green Future strategic theme, the National Institute for Health and Care Research (NIHR), Medical Research Council (MRC), Natural Environment Research Council (NERC) and Engineering and Physical Sciences Research Council (EPSRC) APP29811 MR/Z506692/1. R.F. is an employee of KARL STORZ Endoscopy (UK) Ltd. No employee of KARL STORZ Endoscopy (UK) Ltd was directly involved in the study design or data analysis or had a deciding editorial role in writing the final manuscript. J.B.J. has received teaching and lecturing honoraria from KARL STORZ Endoscopy (UK) Ltd. No other authors have any commercial or financial relationship with KARL STORZ Endoscopy (UK) Ltd, or any other conflicts of interest.

## Funding Statement

The study received no external funding from any source. R.F. is an employee of KARL STORZ Endoscopy (UK) Ltd.

## Supporting information


**Table S1.** Multivariable modelling of factors associated with number of uses prior to failure with flexible cystoscopes returned to the supplier but with no defect detected excluded (*n* = 215).
**Table S2.** Multivariable modelling of factors associated with minutes of use prior to failure.
**Table S3.** Multivariable modelling of factors associated with number of uses prior to failure for subgroups of reasons for failure.

## Data Availability

The data used in this study are commercial and owned by KARL STORZ Endoscopy (UK) Ltd. The authors have no authority to place them in the public domain.
