# Supplementary material for: Factors associated with flexible cystoscope longevity: an analysis of supplier and health service datasets
Source: BJU Int. 2025 Dec 23;137(3):509–18. doi: 10.1111/bju.70133 (PMC12907773; doi:10.1111/bju.70133)
Supplement: Supplementary file 1 — Table S1. Multivariable modelling of factors associated with number of uses prior to failure with flexible cystoscopes returned to the supplier but with no defect detected excluded (n = 215). Table S2. Multivariable modelling of factors associated with minutes of use prior to failure. Table S3. Multivariable modelling of factors associated with number of uses prior to failure for subgroups of reasons for failure. [file BJU-137-509-s001.docx]

**Associations with flexible cystoscope longevity: an analysis of supplier and health service datasets** – **supplementary materials**

**Supplementary material Table S1: Multivariable modelling of factors associated with number of uses prior to failure with flexible cystoscopes returned to the supplier but with no defect detected excluded (n = 215)**

|  | **IRR (95% CI), significance** |
| --- | --- |
| **Type of hospital** |  |
| Private | 1 (reference) |
| NHS | 1.71 (1.38 to 2.12), p < 0.001 |
| **Hospital location** |  |
| England | 1 (reference) |
| Wales | 0.78 (0.56 to 1.09), p = 0.151 |
| Scotland | 1.50 (1.11 to 2.05), p = 0.009 |
| **Year of return to manufacturer** |  |
| 2019 | 1 (reference) |
| 2020 | 1.44 (1.19 to 1.75), p < 0.001 |
| 2021 | 1.54 (1.28 to 1.86), p < 0.001 |
| 2022 | 1.74 (1.44 to 2.11), p < 0.001 |
| 2023 | 1.89 (1.56 to 2.29), p < 0.001 |
| 2024 | 1.74 (1.38 to 2.19), p < 0.001 |
| **Cleaning location** |  |
| Off-site | 1 (reference) |
| On-site and different complex | 1.91 (1.4 to 2.61), p < 0.001 |
| On-site and same complex | 2.49 (1.77 to 3.52), p < 0.001 |
| **Decontamination** |  |
| Dedicated urology decontamination unit | 1 (reference) |
| General endoscopy decontamination unit | 1.16 (0.83 to 1.61), p = 0.389 |
| General hospital sterilisation and decontamination unit | 0.88 (0.6 to 1.29), p = 0.505 |
| **Storage** |  |
| Vacuum packed | 1 (reference) |
| Drying cabinet | 0.88 (0.71 to 1.08), p = 0.219 |
| Bowl and rewashed | 1.55 (1.32 to 1.82), p < 0.001 |
| **On-site endoscopic specialist available** |  |
| No | 1 (reference) |
| Yes | 2.11 (1.52 to 2.93), p < 0.001 |
| **Frequency of staff decontamination training** |  |
| Once per year | 1 (reference) |
| Twice per year | 0.81 (0.68 to 0.96), p = 0.013 |
| More than twice per year | 0.75 (0.62 to 0.91), p = 0.003 |

IRR = incident rate ratio. CI = confidence interval

**Supplementary material Table S2: Multivariable modelling of factors associated with minutes of use prior to failure**

|  | **IRR (95% CI), significance** |
| --- | --- |
| **Type of hospital** |  |
| Private | 1 (reference) |
| NHS | 1.83 (1.49 to 2.24), p < 0.001 |
| **Hospital location** |  |
| England | 1 (reference) |
| Wales | 0.74 (0.53 to 1.04), p = 0.081 |
| Scotland | 1.35 (1.01 to 1.81), p = 0.043 |
| **Year of return to manufacturer** |  |
| 2019 | 1 (reference) |
| 2020 | 1.44 (1.19 to 1.74), p < 0.001 |
| 2021 | 1.55 (1.29 to 1.87), p < 0.001 |
| 2022 | 1.69 (1.41 to 2.04), p < 0.001 |
| 2023 | 1.82 (1.52 to 2.19), p < 0.001 |
| 2024 | 1.69 (1.36 to 2.09), p < 0.001 |
| **Cleaning location** |  |
| Off-site | 1 (reference) |
| On-site and different complex | 1.6 (1.19 to 2.16), p = 0.002 |
| On-site and same complex | 2.36 (1.7 to 3.28), p < 0.001 |
| **Decontamination** |  |
| Dedicated urology decontamination unit | 1 (reference) |
| General endoscopy decontamination unit | 1.43 (1.04 to 1.96), p = 0.029 |
| General hospital sterilisation and decontamination unit | 1.08 (0.75 to 1.55), p = 0.683 |
| **Storage** |  |
| Vacuum packed | 1 (reference) |
| Drying cabinet | 0.90 (0.74 to 1.10), p = 0.303 |
| Bowl and rewashed | 1.37 (1.17 to 1.61), p < 0.001 |
| **On-site endoscopic specialist available** |  |
| No | 1 (reference) |
| Yes | 2.52 (1.77 to 3.58), p < 0.001 |
| **Frequency of staff decontamination training** |  |
| Once per year | 1 (reference) |
| Twice per year | 0.82 (0.69 to 0.96), p = 0.016 |
| More than twice per year | 0.84 (0.70 to 1.01), p = 0.061 |
| **England only additional variable** |  |
| **Annual hospital trust volume** |  |
| 1^st^ (lowest) volume category | 1 (reference) |
| 2^nd^ volume category | 0.71 (0.48 to 1.05), p = 0.084 |
| 3^rd^ volume category | 0.32 (0.24 to 0.41), p < 0.001 |
| 4^th^ volume category | 0.79 (0.59 to 1.06), p = 0.117 |
| 5^th^ (highest) volume category | 0.78 (0.60 to 1.01), p = 0.062 |

IRR = incident rate ratio. CI = confidence interval

**Supplementary material Table S3: Multivariable modelling of factors associated with number of uses prior to failure for subgroups of reasons for failure**

| **Characteristic** | **Damage to working channel** | **Damage to control handle housing** | **Damage to angulation cover** | **Damage to supply plug** | **Poor image quality** | **Damage to adhesive ring** | **Damage to distal head** |
| --- | --- | --- | --- | --- | --- | --- | --- |
|  |  |  |  |  |  |  |  |
| **Type of hospital** |  |  |  |  |  |  |  |
| Private | 1 (reference) | 1 (reference) | 1 (reference) | 1 (reference) | 1 (reference) | 1 (reference) | 1 (reference) |
| NHS | 1.24 (0.87 to 1.77), p = 0.231 | 1.37 (1.02 to 1.84), p = 0.037 | 2.35 (1.91 to 2.9), p < 0.001 | 3.09 (2.26 to 4.24), p < 0.001 | 1.79 (1.31 to 2.46), p < 0.001 | 2.67 (2.00 to 3.57), p < 0.001 | 2.07 (1.68 to 2.56), p < 0.001 |
| **Hospital location** |  |  |  |  |  |  |  |
| England | 1 (reference) | 1 (reference) | 1 (reference) | 1 (reference) | 1 (reference) | 1 (reference) | 1 (reference) |
| Wales | 1.63 (0.78 to 3.39), p = 0.192 | 1.16 (0.72 to 1.87), p = 0.534 | 1.16 (0.85 to 1.59), p = 0.341 | 1.29 (0.84 to 1.98), p = 0.251 | 0.95 (0.55 to 1.65), p = 0.853 | 1.94 (1.01 to 3.73), p = 0.047 | 1.35 (0.98 to 1.84), p = 0.063 |
| Scotland | 1.45 (0.84 to 2.52), p = 0.183 | 1.58 (1.05 to 2.37), p = 0.028 | 1.55 (1.11 to 2.17), p = 0.010 | 1.47 (0.9 to 2.38), p = 0.120 | 1.77 (1.08 to 2.9), p = 0.024 | 0.95 (0.58 to 1.53), p = 0.823 | 1.35 (0.98 to 1.87), p = 0.070 |
| **Year of return to manufacturer** |  |  |  |  |  |  |  |
| 2019 | 1 (reference) | 1 (reference) | 1 (reference) | 1 (reference) | 1 (reference) | 1 (reference) | 1 (reference) |
| 2020 | 0.92 (0.66 to 1.28), p = 0.628 | 0.94 (0.74 to 1.19), p = 0.595 | 1.12 (0.94 to 1.34), p = 0.205 | 1.2 (0.92 to 1.56), p = 0.179 | 1.42 (1.07 to 1.9), p = 0.017 | 1 (0.73 to 1.37), p = 0.991 | 1.22 (1.01 to 1.47), p = 0.043 |
| 2021 | 0.91 (0.67 to 1.25), p = 0.570 | 1.00 (0.79 to 1.26), p = 0.975 | 1.22 (1.03 to 1.46), p = 0.022 | 1.3 (1 to 1.69), p = 0.053 | 1.21 (0.91 to 1.61), p = 0.187 | 1.08 (0.8 to 1.46), p = 0.624 | 1.2 (1 to 1.45), p = 0.053 |
| 2022 | 0.94 (0.63 to 1.40), p = 0.757 | 0.66 (0.49 to 0.89), p = 0.007 | 1.31 (1.07 to 1.60), p = 0.008 | 1.76 (1.31 to 2.34), p < 0.001 | 1.75 (1.26 to 2.42), p = 0.001 | 1.36 (1 to 1.87), p = 0.053 | 1.43 (1.18 to 1.74), p < 0.001 |
| 2023 | 0.49 (0.34 to 0.72), p < 0.001 | 0.43 (0.32 to 0.58), p < 0.001 | 1.40 (1.15 to 1.71), p = 0.001 | 1.98 (1.52 to 2.58), p < 0.001 | 1.45 (1 to 2.1), p = 0.048 | 1.18 (0.82 to 1.69), p = 0.366 | 1.42 (1.17 to 1.73), p < 0.001 |
| 2024 | 0.87 (0.52 to 1.47), p = 0.613 | 0.63 (0.41 to 0.99), p = 0.043 | 1.54 (1.21 to 1.96), p < 0.001 | 1.68 (1.26 to 2.25), p < 0.001 | 2.31 (1.45 to 3.67), p < 0.001 | 1.12 (0.72 to 1.72), p = 0.619 | 1.36 (1.07 to 1.72), p = 0.011 |
| **Cleaning location** |  |  |  |  |  |  |  |
| Off-site | 1 (reference) | 1 (reference) | 1 (reference) | 1 (reference) | 1 (reference) | 1 (reference) | 1 (reference) |
| On-site and different complex | 2.89 (1.28 to 6.55), p = 0.011 | 0.55 (0.28 to 1.08), p = 0.081 | 1.96 (1.31 to 2.91), p = 0.001 | 2.69 (1.54 to 4.71), p = 0.001 | 1.02 (0.44 to 2.36), p = 0.972 | 1.55 (0.94 to 2.56), p = 0.086 | 1.4 (0.94 to 2.08), p = 0.101 |
| On-site and same complex | 4.1 (1.73 to 9.71), p = 0.001 | 0.6 (0.3 to 1.21), p = 0.152 | 2.48 (1.62 to 3.79), p < 0.001 | 3.4 (1.88 to 6.14), p < 0.001 | 1.12 (0.47 to 2.69), p = 0.794 | 1.52 (0.87 to 2.67), p = 0.141 | 1.81 (1.18 to 2.76), p = 0.006 |
| **Decontamination** |  |  |  |  |  |  |  |
| Dedicated urology decontamination unit | 1 (reference) | 1 (reference) | 1 (reference) | 1 (reference) | 1 (reference) | 1 (reference) | 1 (reference) |
| General endoscopy decontamination unit | 2.08 (1.14 to 3.78), p = 0.016 | 1.18 (0.74 to 1.89), p = 0.489 | 1.78 (1.28 to 2.47), p = 0.001 | 1.52 (0.85 to 2.7), p = 0.154 | 1.57 (0.82 to 2.99), p = 0.174 | 1.51 (0.77 to 2.96), p = 0.236 | 2.05 (1.45 to 2.89), p < 0.001 |
| General hospital sterilisation and decontamination unit | 1.89 (0.96 to 3.7), p = 0.065 | 1.15 (0.68 to 1.94), p = 0.601 | 1.35 (0.92 to 1.97), p = 0.12 | 0.93 (0.5 to 1.73), p = 0.828 | 1.1 (0.55 to 2.21), p = 0.787 | 1.71 (0.83 to 3.55), p = 0.147 | 1.57 (1.07 to 2.31), p = 0.022 |
| **Storage** |  |  |  |  |  |  |  |
| Vacuum packed | 1 (reference) | 1 (reference) | 1 (reference) | 1 (reference) | 1 (reference) | 1 (reference) | 1 (reference) |
| Drying cabinet | 1.24 (0.84 to 1.83), p = 0.282 | 1.47 (1.08 to 2), p = 0.014 | 1.08 (0.88 to 1.33), p = 0.440 | 0.94 (0.72 to 1.23), p = 0.645 | 1.02 (0.72 to 1.45), p = 0.903 | 1.23 (0.85 to 1.77), p = 0.268 | 1.14 (0.93 to 1.4), p = 0.206 |
| Bowl and rewashed | 1.79 (1.3 to 2.47), p < 0.001 | 1.68 (1.35 to 2.09), p < 0.001 | 1.4 (1.2 to 1.63), p < 0.001 | 1.18 (0.93 to 1.49), p = 0.179 | 1.29 (0.98 to 1.7), p = 0.068 | 1.04 (0.82 to 1.32), p = 0.76 | 1.34 (1.15 to 1.56), p < 0.001 |
| **On-site endoscopic specialist available** |  |  |  |  |  |  |  |
| No | 1 (reference) | 1 (reference) | 1 (reference) | 1 (reference) | 1 (reference) | 1 (reference) | 1 (reference) |
| Yes | 1.16 (0.5 to 2.7), p = 0.724 | 2.43 (1.53 to 3.88), p < 0.001 | 1.45 (1.05 to 2.01), p = 0.024 | 1.38 (0.88 to 2.16), p = 0.163 | 1.82 (0.99 to 3.37), p = 0.054 | 0.85 (0.45 to 1.59), p = 0.602 | 1.43 (1.04 to 1.97), p = 0.027 |
| **Frequency of staff decontamination training** |  |  |  |  |  |  |  |
| Once per year | 1 (reference) | 1 (reference) | 1 (reference) | 1 (reference) | 1 (reference) | 1 (reference) | 1 (reference) |
| Twice per year | 0.71 (0.51 to 0.99), p = 0.041 | 0.58 (0.46 to 0.74), p < 0.001 | 0.78 (0.66 to 0.92), p = 0.003 | 1.08 (0.82 to 1.41), p = 0.599 | 0.89 (0.65 to 1.21), p = 0.446 | 0.93 (0.71 to 1.22), p = 0.587 | 0.79 (0.67 to 0.94), p = 0.006 |
| More than twice per year | 1.03 (0.68 to 1.55), p = 0.891 | 0.85 (0.63 to 1.13), p = 0.264 | 0.68 (0.56 to 0.84), p < 0.001 | 0.69 (0.52 to 0.92), p = 0.010 | 0.76 (0.51 to 1.14), p = 0.184 | 0.71 (0.52 to 0.97), p = 0.029 | 0.69 (0.56 to 0.85), p < 0.001 |

IRR = incident rate ratio. CI = confidence interval
